# Supplementary material for: Increased theta/alpha synchrony in the habenula-prefrontal network with negative emotional stimuli in human patients
Source: eLife. 2021 Jul 12;10:e65444. doi: 10.7554/eLife.65444 (PMC8275130; doi:10.7554/eLife.65444)
Supplement: Transparent reporting form [file elife-65444-transrepform.docx]

***eLife’s* transparent reporting form**

We encourage authors to provide detailed information *within their submission* to facilitate the interpretation and replication of experiments. Authors can upload supporting documentation to indicate the use of appropriate reporting guidelines for health-related research (see [EQUATOR Network](http://www.equator-network.org/%20)), life science research (see the [BioSharing Information Resource](https://biosharing.org/)), or the [ARRIVE guidelines](http://www.plosbiology.org/article/info:doi/10.1371/journal.pbio.1000412) for reporting work involving animal research. Where applicable, authors should refer to any relevant reporting standards documents in this form.

If you have any questions, please consult our Journal Policies and/or contact us: [editorial@elifesciences.org](mailto:editorial@elifesciences.org).

**Sample-size estimation**

- You should state whether an appropriate sample size was computed when the study was being designed
- You should state the statistical method of sample size computation and any required assumptions
- If no explicit power analysis was used, you should describe how you decided what sample (replicate) size (number) to use

Please outline where this information can be found within the submission (e.g., sections or figure legends), or explain why this information doesn’t apply to your submission:

A previous study with similar paradigm has found beta band activities in the subgenual anterior cingulate cortex were modulated by emotional arousal based on recordings from 8 patients with treatment resistant depression (Huebl et al. 2016).

To our knowledge, this is the first study to investigate the activities in the habenula area and the prefrontal cortex-habenula network in the perception of emotional stimuli in human participants using the direct measurements of local field potentials from the habenula area and simultaneous MEG recordings. This study was made feasible with the unique opportunity of the clinical trial on DBS targeting the habenula as a potential treatment for psychiatric disorders in a center for functional neurosurgery equipped with the MEG device. Therefore, this is an explorative study and we did not perform statistical power analysis for sample size computation. Instead, we tried to recruit all participants who were eligible and well enough after the first surgery for the implantation of the electrodes. In total, we have recorded 9 patients for the study.

Huebl J, Brucke C, Merkl A, Bajbouj M, Schneider GH, Kuhn AA. Processing of emotional stimuli is reflected by modulations of beta band activity in the subgenual anterior cingulate cortex in patients with treatment resistant depression. Social cognitive and affective neuroscience 2016; 11(8): 1290-8.

**Replicates**

- You should report how often each experiment was performed
- You should include a definition of biological versus technical replication
- The data obtained should be provided and sufficient information should be provided to indicate the number of independent biological and/or technical replicates
- If you encountered any outliers, you should describe how these were handled
- Criteria for exclusion/inclusion of data should be clearly stated
- High-throughput sequence data should be uploaded before submission, with a private link for reviewers provided (these are available from both GEO and ArrayExpress)

Please outline where this information can be found within the submission (e.g., sections or figure legends), or explain why this information doesn’t apply to your submission:

1. The experimental paradigm, instructions given to each participant and how the recording was performed are all described in the Materials and Methods.

2. When activities within the habenula were considered, our biological replicates, i.e. “parallel measurements of biologically distinct samples that capture random biological variation” as defined by Blainey et al (2014, Nature Methods), are the 18 recorded habenula from 9 patients. The details of the patients were listed in Table 1, the locations of all electrodes (N=18) are presented in Fig. 2A. When the cortical activities were considered, the biological replicates are the number of patients after excluding the one patient with lots of artefacts in the MEG recordings (N= 8). The number of biological replicates were clearly stated in the results and in the legend for each figure.

3. Excluding/inclusion of data are described in the ‘LFP and MEG Data Analysis’ section of the Materials and Methods. Raw time series of the recordings were visually inspected. For each participant, trials with artifacts shown as flat recording or large amplitude (>5 standard deviation of other recordings) high frequency noise were detected and excluded from analysis. There is one patient with whom the MEG recordings were corrupted with lots of artefact. The MEG recordings from this patient was excluded from further analysis.

Line 406 - 410: All LFP and MEG signals were divided into event-related epochs aligned to the stimuli onset (-2500 to 4500 ms around the stimulus onset) and visually inspected for artefacts due to movement and other interferences. Trials with artefacts were removed from final analysis, leaving a mean number of 27 trials (range 18-30) for each valence category for each subject.

Line 383 - 387: Eye blink and heartbeat artefacts in the MEG signals were identified by ICA and the low frequency, high amplitude components were removed from all MEG sensors. The MEG data of one subject (case 4) had to be discarded due to severe artefacts across all MEG channels. Hence, all reported results with MEG data are based on eight subjects.

**Statistical reporting**

- Statistical analysis methods should be described and justified
- Raw data should be presented in figures whenever informative to do so (typically when N per group is less than 10)
- For each experiment, you should identify the statistical tests used, exact values of N, definitions of center, methods of multiple test correction, and dispersion and precision measures (e.g., mean, median, SD, SEM, confidence intervals; and, for the major substantive results, a measure of effect size (e.g., Pearson's r, Cohen's d)
- Report exact p-values wherever possible alongside the summary statistics and 95% confidence intervals. These should be reported for all key questions and not only when the p-value is less than 0.05.

Please outline where this information can be found within the submission (e.g., sections or figure legends), or explain why this information doesn’t apply to your submission:

1. We used non-parametric cluster-based permutation test for the main results (Fig. 3-6) with a cluster threshold of 0.05. Therefore, for those analysis, we just reported p<0.05. For post-hoc analysis with paired t-test, the exact p values were reported (Fig. 3G-H).

Line 441 - 453: A non-parametric cluster-based permutation approach (Maris and Oostenveld, 2007) was applied to normalized time-frequency matrices to identify clusters (time window and frequency band) with significant differences in the power changes induced by the presentation of pictures of different emotional valence. To achieve this, the original paired samples were randomly permuted 1000 times such that each pair was maintained but its assignment to the condition (negative or positive) may have changed to create a null-hypothesis distribution. For each permutation, the sum of the z-scores within suprathreshold-clusters (pre-cluster threshold: p < 0.05) was computed to obtain a distribution of the 1000 largest suprathreshold-cluster values. If the sum of the z-scores within a suprathreshold-cluster of the original difference exceeded the 95th percentile of the permutation distribution, it was considered statistically significant. The average powers in the determined frequency band and time window identified by the cluster-based permutation method between different valence conditions were further compared using post-hoc paired t-test.

2. For the linear mixed effect modelling, the estimated mean value and standard error, and the p values of the fixed effects, as well as the R^2^ value of each model are all presented in Table 2.

3. For source localization based on MEG sensor level recordings, we used a frequency domain beamforming approach. Statistical significance at group level (statistics over subjects) was determined using cluster-based non-parametric permutation testing. A one-tailed (only power increment effect or coherence increment effect) dependent-sample t-test was applied to statistically quantify the differences in neural DICS theta/alpha source power or source coherence between negative and positive emotional stimuli. Therefore, the source areas with significant difference across subjects at a cluster level of 0.05 were identified. For source peaks, the t-values and p-values were reported in the Results.

Line 422 - 424: The dynamic imaging of coherent sources (DICS) beamformer in SPM8 with a single-shell forward model was used to generate maps of the source power difference between conditions on a 5 mm grid co-registered to MNI coordinates.

Line 454 - 457: A one-tailed dependent-sample t statistics and cluster-based permutation testing was applied to statistically quantify the differences in DICS source for power or source coherence between negative and positive emotional stimuli.

(For large datasets, or papers with a very large number of statistical tests, you may upload a single table file with tests, Ns, etc., with reference to sections in the manuscript.)

**Group allocation**

- Indicate how samples were allocated into experimental groups (in the case of clinical studies, please specify allocation to treatment method); if randomization was used, please also state if restricted randomization was applied
- Indicate if masking was used during group allocation, data collection and/or data analysis

Please outline where this information can be found within the submission (e.g., sections or figure legends), or explain why this information doesn’t apply to your submission:

This was not a clinical study. We used a within-subject designed with each participant performed the passive figure viewing task including figures of all of the three valence conditions: positive, neutral and negative. Within-subject comparisons were performed to investigate the effect of valence.

**Additional data files (“source data”)**

- We encourage you to upload relevant additional data files, such as numerical data that are represented as a graph in a figure, or as a summary table
- Where provided, these should be in the most useful format, and they can be uploaded as “Source data” files linked to a main figure or table
- Include model definition files including the full list of parameters used
- Include code used for data analysis (e.g., R, MatLab)
- Avoid stating that data files are “available upon request”

Please indicate the figures or tables for which source data files have been provided:

We have uploaded the data and the code with which one can generate the figures in the main manuscript, as well as the results in Table II.
